# Deep-learning segmentation to select liver parenchyma for categorizing hepatic steatosis on multinational chest CT

**DOI:** 10.1038/s41598-024-62887-2

**Published:** 2024-05-25

**Authors:** Zhongyi Zhang, Guixia Li, Ziqiang Wang, Feng Xia, Ning Zhao, Huibin Nie, Zezhong Ye, Joshua S. Lin, Yiyi Hui, Xiangchun Liu

**Affiliations:** 1 Department of Nephrology, Multidisciplinary Innovation Center for Nephrology, The Second Hospital of Shandong University, Shandong University, Jinan, 250033 Shandong China; 2 Department of Nephrology, Shenzhen Third People’s Hospital, the Second Affiliated Hospital of Southern University of Science and Technology, Shenzhen, 518112 Guangdong China; 3 Department of Nephrology, The First Affiliated Hospital of Hainan Medical University, Haikou, 570102 Hainan China; 4 Department of Cardiovascular Surgery, Wuhan Asia General Hospital, Wuhan, 430000 Hubei China; 5 The First Clinical Medical School, Shanxi Medical University, Taiyuan, 030001 Shanxi China; 6 Department of Nephrology, Chengdu First People’s Hospital, Chengdu, 610021 Sichuan China; 7 Independent Researcher, Boston, MA 02115 USA; 8 Keck School of Medicine, University of Southern California, Los Angeles, CA 90033 USA; 9 Department of Medical Imaging, Shandong Provincial Hospital Affiliated to Shandong First Medical University, Jinan, 250021 Shandong China

**Keywords:** Computed tomography, nnU-Net, Deep learning, Hepatic steatosis, Biomedical engineering, Image processing, Machine learning

## Abstract

Unenhanced CT scans exhibit high specificity in detecting moderate-to-severe hepatic steatosis. Even though many CTs are scanned from health screening and various diagnostic contexts, their potential for hepatic steatosis detection has largely remained unexplored. The accuracy of previous methodologies has been limited by the inclusion of non-parenchymal liver regions. To overcome this limitation, we present a novel deep-learning (DL) based method tailored for the automatic selection of parenchymal portions in CT images. This innovative method automatically delineates circular regions for effectively detecting hepatic steatosis. We use 1,014 multinational CT images to develop a DL model for segmenting liver and selecting the parenchymal regions. The results demonstrate outstanding performance in both tasks. By excluding non-parenchymal portions, our DL-based method surpasses previous limitations, achieving radiologist-level accuracy in liver attenuation measurements and hepatic steatosis detection. To ensure the reproducibility, we have openly shared 1014 annotated CT images and the DL system codes. Our novel research contributes to the refinement the automated detection methodologies of hepatic steatosis on CT images, enhancing the accuracy and efficiency of healthcare screening processes.

## Introduction

Hepatic steatosis, or fatty liver disease, is a pathological condition where intrahepatic fat is equal to or greater than 5% of liver weight^1^. This condition increases the risk of liver cirrhosis, end-stage liver failure, and early mortality^2^. Currently, liver biopsy is the diagnostic standard for hepatic steatosis, but this tool is limited due to the invasive nature and morbidity risks^3^. Noninvasive techniques are widely applied to address this limitation, such as ultrasound (US), magnetic resonance imaging (MRI), and computed tomography (CT)^4^.

While MRI is a non-invasive preference^5–8^, it's worth noting that unenhanced CT has been shown to have linear equivalence in measuring liver fat^9,10^. Therefore, unenhanced CT has emerged as a feasible alternative, particularly for detecting moderate-to-severe steatosis^11^. Among imaging coverages, chest CT has gained significant value for assessing liver fat due to its widespread availability and frequent utilization^12–15^. For example, unenhanced chest CT is highly practical in existing images of lung-cancer screenings and COVID-19 patients, especially in cases where abdominal CT is not even available^13,16–18^.

Researchers have established various indicators to assess hepatic steatosis on CT images, including the liver-spleen attenuation ratio, liver-spleen attenuation difference, and the thresholding of liver attenuation alone^19–22^. Notably, a liver attenuation threshold of ≤ 40 Hounsfield units (HU) can serve as an independent indicator^13,21–23^. The liver attenuation is measured by radiologists on a circular region of interest (ROI) to represent the whole-liver fat content^24^. However, this measurement requires significant time and expertise for population-based studies, posing challenges for incidental assessments and clinical interactions involved in liver diseases. Considering the prevalence of hepatic steatosis, millions of individuals at risk may remain undetected^17,25^. Therefore, an automated tool becomes an urgent necessity for identifying these potential patients in large-scale clinical research.

Deep learning (DL) has emerged as a promising tool in the automated analysis of CT images^26–30^. Notably, its potential has been demonstrated in the realms of automated liver segmentation and steatosis assessments^16,30–34^. However, these approaches have not adequately tackled the following challenges in a comprehensive manner^35^. These challenges include (i) developing a fully automated end-to-end process, (ii) achieving accuracy that is comparable to that of an experienced radiologist, (iii) showing generalizability across multinational populations, (iv) providing interpretability to clinicians and patients, and (v) ensuring reproducibility by sharing the DL system and manual annotations. Therefore, our study aims to address all of these challenges by developing a DL system for the purpose of incidentally categorizing hepatic steatosis on existing CT images.

## Results

### Patient characteristics

**Figure 1:**
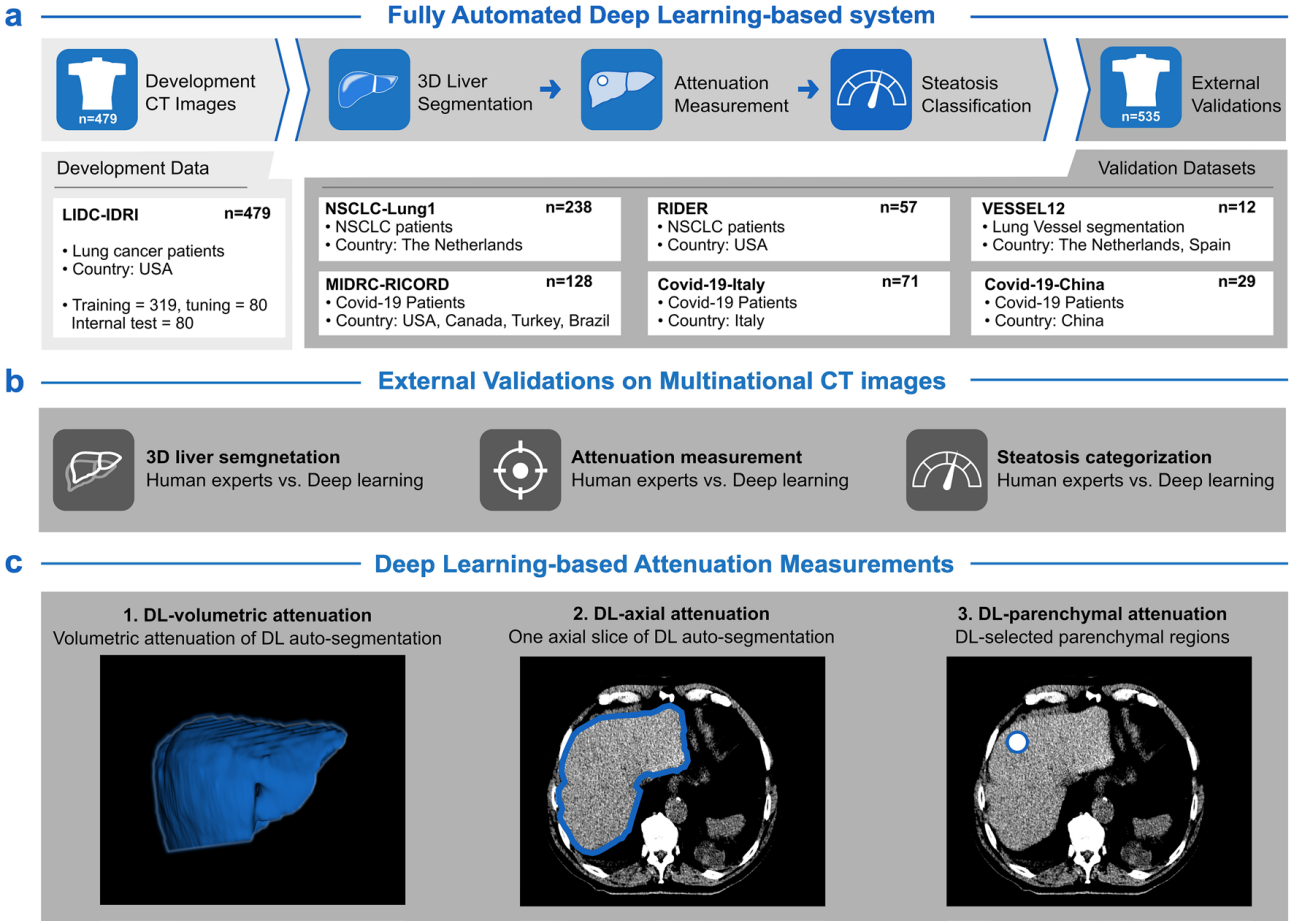
Study design. (**a**) A fully automated deep learning-based system was designed for measuring live attenuation and assessing moderate-to-severe steatosis. The system consists of three steps: 3D liver segmentation, attenuation measurement, and steatosis classification. The development data consisted of 479 CT images from the LIDC-IDRI dataset, which were split into 4:1:1 for training, tuning, and internal testing, respectively. External validations were conducted on a total of 535 CT images from six datasets across eight countries. (**b**) The performance of the deep learning system was evaluated using radiologist-validated ground truths. (**c**) Three deep learning-based methods were compared to measure CT attenuation in Hounsfield units (HU). They are (1) DL-volumetric, which measured attenuation volumetrically; (2) DL-axial, which measured attenuation on a single axial slice containing the largest cross-sectional area; and (3) DL-parenchymal, which measured attenuation on parenchymal portions selected on axial slices using 3D auto-segmentation. Figure c displays examples of DL-based attenuation measurements. *DL* deep learning, *HU* Hounsfield unit.

An overview of the study design is provided in Fig. 1. This retrospective study proposed a novel DL-parenchymal method for accurate identification of moderate-to-severe hepatic steatosis. 1014 CT images were included of 986 adult participants across eight countries (Figure S1). The mean liver attenuation was 56.33 ± 11.69 across all cohorts. Moderate-to-severe hepatic steatosis was observed in 8.19% of CT images (Table 1).

**Table 1:** Patient characteristics.

Dataset	Partition	Patients	CT images	Steatosis CT	CT attenuation
LIDC-IDRI	Development	479	479	4.59%	60.80 ± 10.32
Lung1	External test	238	238	10.92%	48.96 ± 9.62
RIDER	External test	29	57	3.51%	61.16 ± 13.59
VESSEL12	External test	12	12	0.00%	57.15 ± 7.00
MIDRC-RICORD	External test	128	128	16.41%	52.46 ± 11.35
Covid-19-Italy	External test	71	71	15.49%	53.28 ± 11.45
Covid-19-china	External test	29	29	3.45%	57.55 ± 8.29

This retrospective study comprises 1,014 computed tomography (CT) images of 986 participants across eight countries. The mean and standard deviation of the ground-truth liver attenuation are reported. Moderate-to-severe hepatic steatosis is classified based on ground-truth liver attenuations. The LIDC-IDRI dataset, consisting of 479 CT images, was used as the development data. External validation was conducted on a total of 535 CT images from six other datasets across eight countries.

### Deep learning performance on 3D liver segmentation

**Figure 2:**
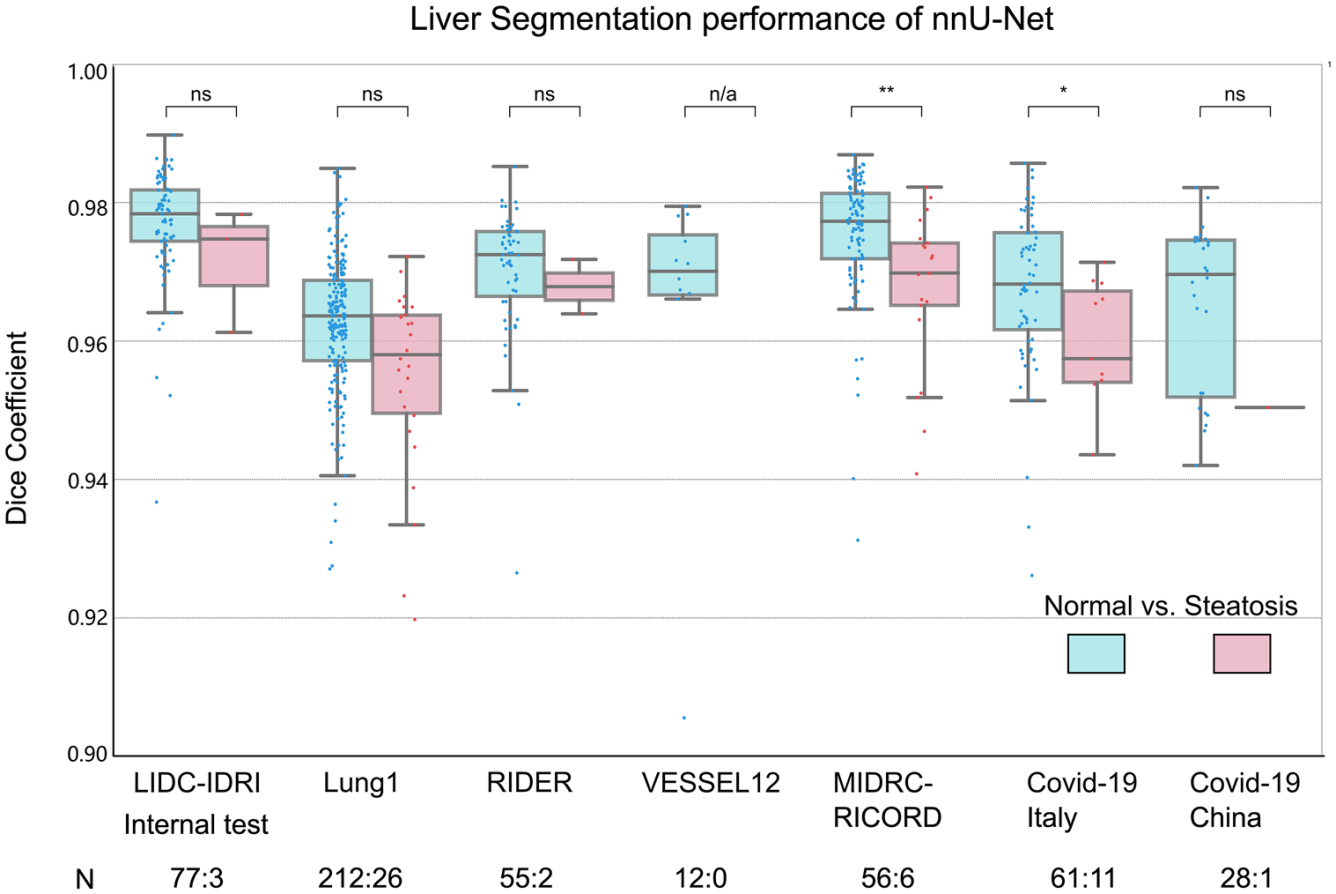
Deep learning segmentation performance. DSC indicates the similarity between expert and DL segmentations. The DSC is also ground by different conditions of the liver: “Normal vs. Steatosis”. The number of CT images is denoted at the bottom (normal vs. steatosis). The result shows that DL achieves an overall high segmentation accuracy, and slightly better performance on the normal liver than that of steatosis. DL = deep learning, DSC = dice similarity coefficient. ***, *p* < 0.001; **, *p* < 0.01; *, *p* < 0.05.

A nnU-Net model was developed and externally validated for liver segmentation. The nnU-Net model achieved a DSC of 0.977 ± 0.008 on the internal test and an average DSC of 0.965 ± 0.020 on external tests (Table 2). Notably, 91.87% of the segmentation results achieved a DSC >  = 0.95. However, a slight decrease in DSC was observed in the presence of hepatic steatosis in each test dataset (Fig. 2). Liver attenuation weakly correlated with the DSC score, as indicated by a Spearman correlation coefficient of 0.293 (Figure S2a). Despite the slight decrease, DSC remained statistically comparable in three out of five test datasets (*p* > 0.050, Fig. 2). Segmentation examples and outlier analysis could be found in Supplementary Text 4 and Figure S3.

**Table 2:** Liver segmentation performance of nnU-Net vs. 3D U-Net.

Test set	AI model	DSC	JC	ASSD (mm)	HD (mm)
Internal set (n = 80)	nnU-Net	**0.977 ± 0.008**	**0.954 ± 0.015**	**0.536 ± 0.292**	**13.049 ± 8.027**
	3D U-Net	0.973 ± 0.014	0.947 ± 0.026	0.686 ± 0.529	16.760 ± 10.719
External set (n = 535)	nnU-Net	**0.965 ± 0.020**	**0.932 ± 0.034**	**1.101 ± 2.551**	**19.967 ± 34.473**
	3D U-Net	0.940 ± 0.050	0.891 ± 0.085	1.791 ± 3.029	26.993 ± 34.636
	3D U-Net*	0.947 ± 0.060	0.904 ± 0.091	1.459 ± 2.769	27.138 ± 34.822

This table presented a comparative analysis of segmentation accuracy achieved by two AI models developed in this study, namely nnU-Net and 3D U-Net. In addition, the performance of a 3D U-Net model without retraining was also included for comparison on external tests. All metrics were presented in mean value with standard deviation.

*DSC* Dice similarity coefficient, *JC* Jaccard coefficient, *HD* Hausdorff distance, *ASSD* average symmetric surface distance.

Significant values are in bold.

In comparison to the nnU-Net, 3D U-Net models showed significantly lower DSC on external tests, which was 0.940 ± 0.050 with retraining and 0.947 ± 0.060 without retraining, respectively (Table 2). The Spearman correlation coefficient for the 3D U-Net model was 0.537, indicating that its performance was more influenced by the liver attenuation and/or steatosis conditions (Figure S2b).

### Deep learning performance on liver attenuation

**Figure 3:**
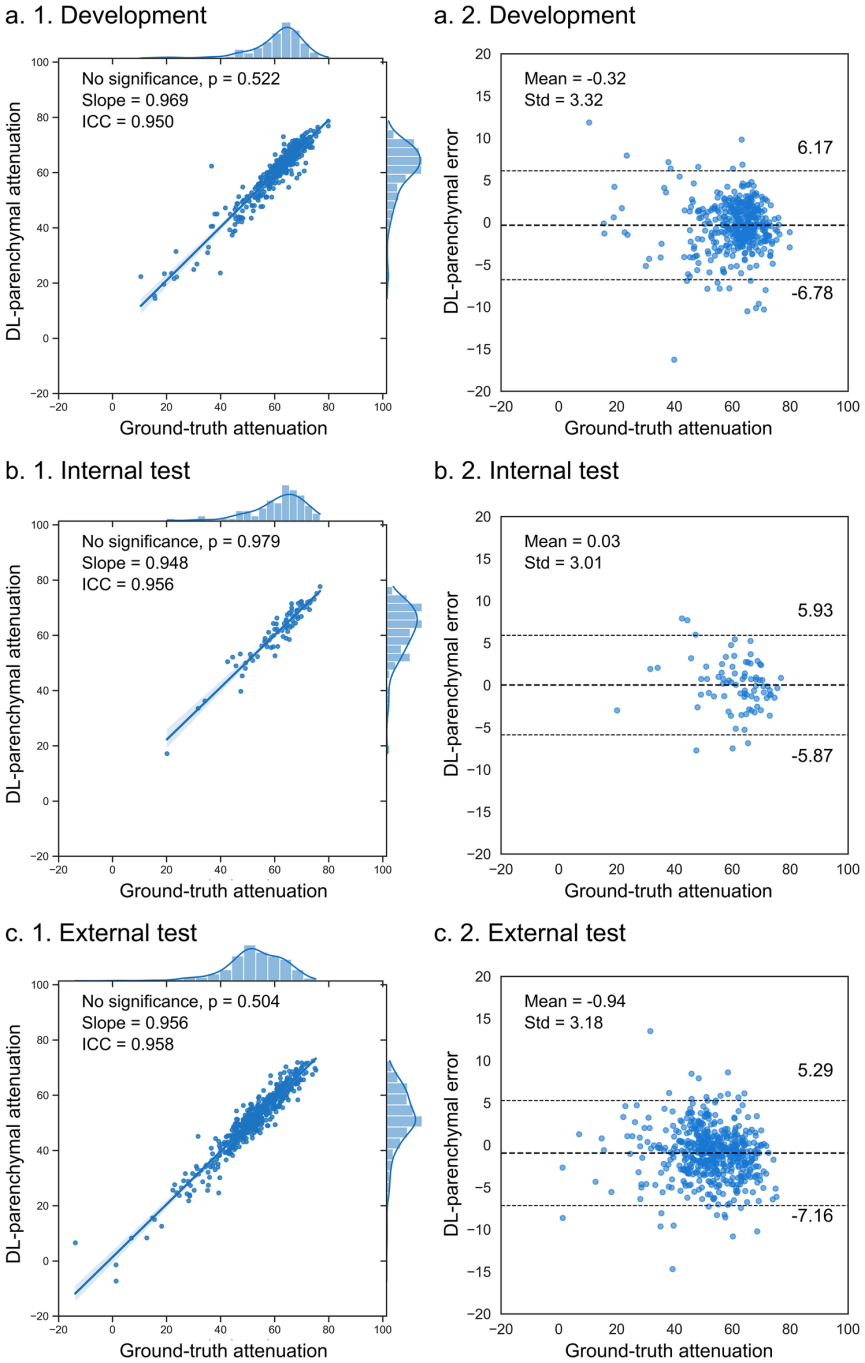
Concordances of DL-parenchymal vs. ground-truth attenuation. DL-parenchymal attenuation was compared to ground-truth attenuations on partitioned datasets (development set: n = 399, internal test set: n = 80, external test set: n = 535). The scatter plot shows the p-value, slope, and Spearman correlation coefficient. The agreement was further assessed using Bland–Altman analysis, with liver attenuations in Hounsfield units (HU) on the x-axis. The bold line represents the actual mean difference (error), and the other two dotted lines show 95% limits of agreements.

**Figure 4:**
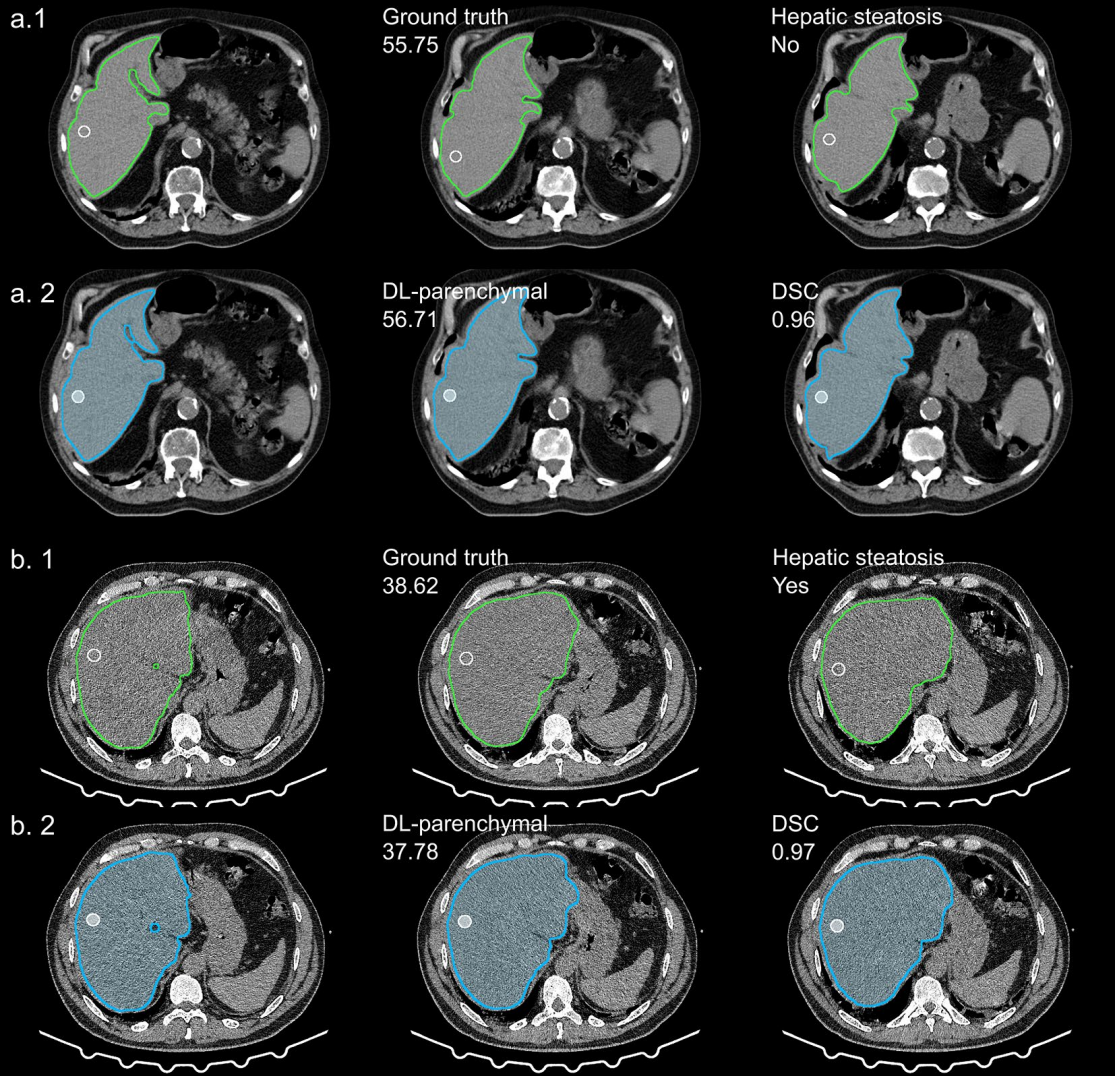
Examples of DL-parenchymal vs. ground-truth attenuation. Two examples presented the portion placement of DL-parenchymal attenuation. In Figure a. 1, manual segmentation and selection were used to measure the liver attenuation of a no steatosis condition. In contrast, DL auto-segmentation and DL-selected parenchymal regions were used for attenuation measurements in Figure a. 2. Another example of the liver with steatosis was shown in Figures b. 1 and b. 2. *DL* deep learning, *DSC* Dice similarity coefficient.

Three DL-based methods were evaluated for their accuracy for measuring liver attenuation. As depicted in Table 3, both the DL-volumetric and DL-axial attenuations were significantly different from the reference standards in the development and external test sets (*p* < 0.050). In contrast, the DL-parenchymal attenuation achieved no significant difference in all partitioned sets (*p* > 0.050, Fig. 3 and Figure S4). In external tests, parenchymal ICC was 0.958 (95% CI, 0.944–0.967) and the MSE was 2.432, indicating the external generalizability of the DL-parenchymal method. Examples of DL-parenchymal attenuation were shown in Fig. 4, and the outliers were analyzed in Supplementary Text 5 and Figure S5.

**Table 3:** Deep learning-based attenuation vs. ground-truth attenuation.

Attenuation	Data partition	DL-parenchymal	DL-volumetric	DL-axial
P value	Development	**p = 0.522**	p < 0.001	p < 0.001
	Internal test	**p = 0.979**	p = 0.173	p = 0.438
	External test	**p = 0.504**	p = 0.013	p = 0.022
MSE	Development	**2.328**	2.563	2.477
	Internal test	**2.291**	2.385	2.389
	External test	**2.432**	2.517	2.616
ICC	Development	**0.950 95% CI** **0.939–0.959**	0.923 95% CI 0.842–0.955	0.949 95% CI 0.885–0.972
	Internal test	**0.956 95% CI** **0.932–0.972**	0.955 95% CI 0.912**–**0.975	0.955 95% CI 0.924**–**0.972
	External test	**0.958 95% CI** **0.944–0.967**	0.957 95% CI 0.911–0.975	0.950 95% CI 0.927–0.964

This table presented the accuracy of DL-based attenuation on partitioned datasets. The DL-volumetric and DL-axial attenuation were derived from manual segmentation for the development set. For the internal and external test sets, these values were obtained through DL auto-segmentation. Evaluation metrics were the p value, MSE, and ICC with 95% CI.

*DL* deep learning, *MSE* mean absolute error, *ICC* intraclass correlation coefficient, *CI* confidence interval.

Significant values are in bold.

### Deep learning performance on steatosis classification

In the third step, a threshold of DL-based attenuation < 40 HU was applied to the automated categorization of moderate-to-severe hepatic steatosis. On the internal test set, the DL-parenchymal category achieved an AUC of 0.994 (95% CI, 0.980–1.000), a sensitivity of 1.000 (95% CI, 1.000–1.000), and a specificity of 0.987 (95% CI, 0.960–1.000). On the external test set, it achieved an AUC of 0.942 (95% CI, 0.899—0.975), a sensitivity of 0.902 (95% CI, 0.815—0.966), and a specificity of 0.989 (95% CI, 0.980—0.998). DL-parenchymal outperformed the other two DL-based methods, achieving the highest AUC and sensitivity on external tests. The DL-based classifications were summarized in Table S2, and confusion matrices are presented in Figure S6.

### Deep learning vs. multiple human experts for measuring attenuation

**Figure 5:**
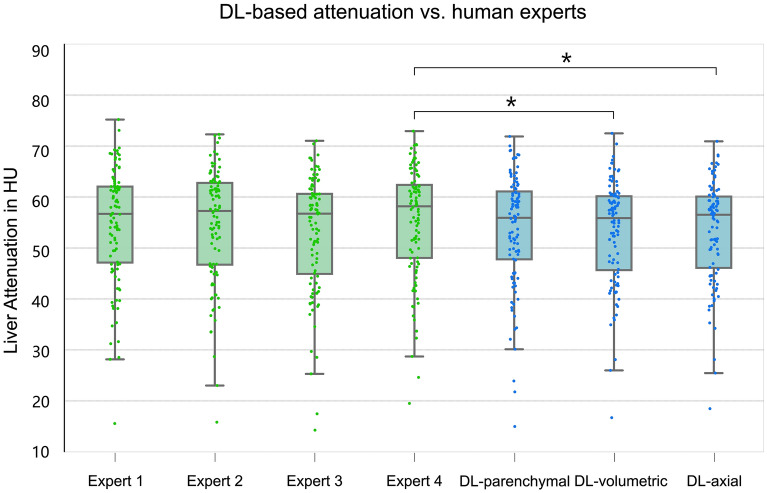
Attenuation concordance of multiple human experts and DL-based methods. A subset of 100 images was independently measured by four human experts and DL-based methods. Significant differences are shown with denoted p values: ***, *p* < 0.001; **, *p* < 0.010; *, *p* < 0.050. *DL* deep learning.

Figure 5 illustrated the comparison between the performance of DL-based methods and that of four human experts in measuring liver attenuation. It was the DL-parenchymal attenuation that achieved statistically comparable results to those of four human experts (*p* > 0.050), indicating its independence of inter-reader variability. However, both DL-volumetric and DL-axial methods showed a significant difference against one human expert (*p* < 0.050). Moreover, the manual measurements of all four human experts were statistically the same (*p* > 0.050).

## Discussion

This study aimed to assess the feasibility of fully automated categorizations for hepatic steatosis using unenhanced chest CT images. Our findings demonstrated that DL-measured parenchymal attenuation exhibited both radiologist-level accuracy and cross-national generalizability. Additionally, our study provided open access to DL algorithms and radiologist-validated segmentations, which can contribute to the advancement of biomedical segmentation algorithms. The availability of this open-source DL system could promote incidental assessments of hepatic steatosis, ultimately enhancing clinical understanding and management of the complex interactions involved in liver diseases.

One primary concern is the clinical value of our automated tool. While the liver biopsy is the current diagnosis standard, its invasiveness and morbidity risk make it impractical for large-scale studies^3^. Although MRI is a non-invasive preference^5^, non-enhanced CT scans have been shown to be linearly correlated with MRI and highly specific for detecting moderate-to-severe steatosis conditions^10,22,36,37^. Furthermore, unenhanced chest CT is increasingly being ordered in lung screening cohorts and for COVID-19 patients, making it more practical than abdominal CT for population-based incidental assessments^12,13,15,38,39^. For instance, annual lung cancer screening is recommended for long-term smokers in America and China, potentially involving millions of individuals undergoing unenhanced chest CT scans^17,25,40^. Although several studies^19,20,41^ proposed alternate indicators such as the liver-spleen attenuation ratio and the liver-spleen attenuation difference, unenhanced attenuation alone already offers high specificity for CT-based assessments^21,22^.

Consequently, the clinical value of our DL method lies in its ability to automate the assessment of moderate-to-severe hepatic steatosis on existing unenhanced chest CT images. A previous study observed a relatively high proportion of patients experiencing severe COVID-19 infections with DL-classified hepatic steatosis^30^. However, this automated method was solely validated on COVID-19 patients, and as a result, its generalizability is limited. Other studies have investigated the associations between hepatic steatosis and increased risks of cardiovascular disease and all-cause mortality^42,43^. However, these investigations have relied on laborious manual measurements. Hence, our method provides automated solutions for incidental assessments of hepatic steatosis on multinational populations. This makes it possible to explore associations with disease severity and increased risks on a broader scale, such as in lung cancer screening cohorts and COVID-19 patients.

The first novelty of our DL method lies in its ability to achieve expert-level accuracy in the automated selection of parenchymal regions for measuring liver attenuation. While other studies have explored DL methods for measuring liver attenuation, their accuracy was often limited by the inclusion of non-parenchymal regions within the whole-liver attenuation^16,30,33^. For instance, one previous study investigated the relationship between COVID-19 risk and both slice-wise and whole-liver attenuation assessment^30^, which is equivalent to our DL-axial and DL-volumetric methods. We have compared these methods with the ground truth of the 2D-ROI manual approach, revealing statistical differences and highlighting their limitations. Another study employed a DL method to automatically select periphery ROIs, but the results lacked statistical comparability with manual measurements supported by a numerical P value^34^. Additionally, its ROI localization involved locating the center point of the liver and morphological operations to determine ROI center. This approach could impose an increase of computation time and complexity, which could be a disadvantage when processing a large dataset. Due to the imperfections in its statistical test and the multi-step ROI process, our DL-parenchymal method demonstrates a more reliable and simplified selection of ROI locations.

The second novelty lies in the generalizability and robustness of liver segmentation. Unlike prior studies that often lack large-scale external validations^16,32^, our deep learning segmentation consistently achieves nearly excellent accuracy across diverse centers and nations. While there is a slight decrease in accuracy in the presence of hepatic steatosis, likely due to the lower radio intensity of fatty liver and an imbalance in the development data, the majority of cases still exhibit nearly excellent segmentation accuracy. This underscores the remarkable robustness of the model despite the challenges posed by hepatic steatosis. The robustness is also evident in our DL-parenchymal attenuation measurements, as they not only align with the accuracy of human readings but also effectively address inter-reader variability. The third novelty is the open accessibility. We have made both DL algorithms and expert segmentations freely available to the public, constituting the most extensive open-sourced liver segmentations on unenhanced chest CT images across centers and nations.

Several limitations should be recognized within our study. Firstly, unenhanced CT scans alone may not be sufficient in detecting mild or coexisting hepatic steatosis, and the use of CT scans also poses radiation exposure concerns. However, we minimized this limitation by assessing moderate-to-severe conditions using existing CT scans that were originally obtained for lung-disease indications. Secondly, manual annotations were conducted by several human experts but validated by only one experienced radiologist, and not all chest CT scans showed the entire liver. Thirdly, the 2D ROI-based approach, despite its proven clinical value, may encounter challenges such as limited coverage and sampling errors. Further investigation is recommended into the correlation of whole-liver attenuation. Fourthly, our DL system, while being open-source, is not yet suitable for medical use due to the absence of rigorous clinical validations and user-friendly graphical interfaces that are essential for direct deployment in clinical settings. Lastly, our DL system lacks prognostic value due to the absence of patient demographics in open-source databases. Future research is needed to address these limitations.

In conclusion, our study presents a DL-based method utilizing a novel DL-parenchymal method for accurate identification of moderate-to-severe hepatic steatosis. By excluding non-parenchymal portions, our method overcomes previous limitations and achieves radiologist-level accuracy with cross-national generalizability. Our DL method could improve incidental assessments of moderate-to-severe hepatic steatosis from multinational chest CT scans, thereby aiding researchers to further explore the complex interactions involved in liver diseases.

## Methods

This retrospective study aimed to assess the feasibility of a novel DL-parenchymal method for accurate identification of moderate-to-severe hepatic steatosis on chest CT images. An overview of the study design is provided in Fig. 1. During the preparation of this work the authors used ChatGPT in order to polish the writing. After using this tool/service, the authors reviewed and edited the content as needed and take full responsibility for the content of the publication. This retrospective study was conducted in accordance with the Declaration of Helsinki guidelines.

Our study did not require Institutional Review Board (IRB) oversight since our materials were sourced from publicly available and deidentified data. Research involving such datasets does not necessitate IRB review if the data are sourced from publicly accessible repositories and are appropriately deidentified, in accordance with regulations set forth by the U.S. Department of Health and Human Services^44,45^. The public datasets used in our study were initially released within the publications: LIDC-IDRI^46^, NSCLC-Lung1^47^, RIDER^48^, VESSEL12^49^, MIDRC-RICORD^50^, COVID-19-Italy^51^, and COVID-19-China^52^. Each dataset underwent deidentification and received approval from its respective licensing committees and/or institutions, accompanied by informed consent from all subjects. Further details, including licenses and download links for each dataset, are available in Supplementary Text 1. Subsequently, chest CT images were extracted from these public datasets, and our team conducted manual liver segmentation to facilitate the development of our AI methods.

### Unenhanced chest CT datasets

The study included 1,014 CT images from seven databases: LIDC-IDRI^46^, NSCLC-Lung1^47^, RIDER^48^, VESSEL12^49^, MIDRC-RICORD^50^, COVID-19-Italy^51^, and COVID-19-China^52^. Three datasets^46–48^ were initially obtained for lung cancer diagnosis, three^50–52^ were initially obtained to diagnose COVID-19, and one^49^ was for lung vessel segmentation research. The cross-national inclusion of these CTs aimed to encompass a heterogeneous distribution of imaging data, as they were acquired using diverse scanners with varying parameters.

The unenhanced chest CT image was the only inclusion criterion of our study, and the following were thus the exclusion criteria. (i) Contrast-enhanced chest CT images, which were selected out by an automatic method^53^ and then validated by a radiologist. (ii) Stomach with contrast agent. (iii) Imaged liver with severe artifacts; livers with tumors or cysts collectively bigger than 5 cm^3^. The consort diagram was shown in Figure S1. Additional details on each dataset could be found in Table S1 and Supplementary Text 2. All CT images were reformatted to the Neuroimaging Informatics Technology Initiative (NIfTI) format, and linear interpolations were employed to resample them to a consistent voxel spacing of 0.7 × 0.7 × 2.5 mm/pixel.

### Manual segmentation and manual measurements of liver attenuation

Imaged livers were manually segmented and the attenuations were manually measured on all of 1,014 CT images. Manual segmentations of liver images were obtained by manually tracing contour lines on axial slices with a thickness of 2.5 mm using 3D Slicer software v5. The reference standard of liver attenuation was measured on parenchymal portions while avoiding areas of heterogeneity, such as hepatic veins, bile ducts, and focal nodules. The selected region had an approximate area of 2 cm^2^, based on the area setting used in previous studies^19,22^.

Initially, manual segmentations and attenuation measurements were initially performed by one data scientist and six clinicians with varying levels of expertise. The clinicians team included a pulmonologist, a cardiovascular surgeon, and four nephrologists with 1, 10, 10, 10, 10, and 15 years of experience, respectively. All manual annotations were subjected to validations and potential corrections by an experienced radiologist (10 years of experience). The annotations that passed the validation process by the radiologist were considered as the reference standard.

To evaluate the inter-reader variability of attenuation measurements, four human experts independently measured liver attenuation for a subset of 100 CT images for an analysis of the manual selections of ROI. Human experts for attenuation measurements underwent thorough training from professional radiologists, specifically on selecting ROIs at the periphery to minimize non-parenchymal heterogeneities. This specialized training ensured their proficiency matches that of expert radiologists and qualified them as professionals on this particular task for a reliable assessment of inter-reader variability. The 100 CT images were chosen as the complete set of images from two external validation datasets: Covid-19-Italy and Covid-19-China.

### Deep learning for 3D liver segmentation

The first step of our DL-based system is a nnU-Net model for automated liver segmentation. The nnU-Net (no-new-Net) refers to a self-adapting DL framework for medical image segmentation^54^. A brand new nnU-Net model weight was trained using a Linux workstation (ubuntu 18.04) with an RTX 3060 GPU using Pytorch^55^. Training epochs were 1000 to find an optimum model weight, with an initial learning rate of 0.01. The loss function used was Dice and cross-entropy loss. The segmented liver was designated as the largest connected component by the connected-component analysis. In addition, a 3D U-Net^33^ model was implemented with or without retraining for performance comparison (Supplementary Text 3).

The development data consisted of 479 CT images from the LIDC-IDRI set, which were randomly split in a ratio of 4:1:1 for training, tuning, and internal testing purposes. External validations were performed on a total of 535 CT images from the remaining six data sets across eight countries, as depicted in Fig. 1a.

### Deep learning to select liver parenchyma for attenuation measurement

The second step of the DL-based automated method involves three distinct types of attenuation measurements: DL-volumetric, DL-axial, and DL-parenchymal attenuation. The DL-volumetric attenuation method calculates the mean voxel value of the 3D auto-segmentation, enabling the computation of an average liver attenuation measure across the entire liver volume. In contrast, the DL-axial attenuation method focuses on a single axial image, specifically the largest slice within the cross-sectional segmentation area. DL-axial attenuation is determined from this selected slice, providing a slice-wise measurement.

The DL-parenchymal method aimed at automatically replicating the process of manual measurements. Specifically, this method utilizes 3D liver segmentation to automatically select ROI portions on the liver parenchyma for the purpose of measuring liver attenuation. For each of the three largest axial slices, a single region per slice is chosen, located at an axial distance of 0.5 cm. The selected region encompasses an approximate area of 2 cm^2^, as determined by the area setting employed in a previous study^19,22^.

The circular ROI is positioned around the leftmost side of the liver, maintaining a 2 cm distance to the right of the leftmost pixel of the liver edge. This strategic placement is empirically designed in accordance with previous studies^13,23,24^. Placing the ROI center close to the leftmost margin ensures the selection of liver parenchyma, effectively avoiding non-parenchymal regions that are typically located towards the center or the opposite side of the liver^21^. The specific distance parameter of 2 cm allows a margin of ~ 1 cm between the ROI and liver contour, which was designed to avoid scenarios where an over-segmented liver might lead to the ROI falling outside the liver region. Examples of DL-measured attenuation were shown in Fig. 1c.

### Statistical analysis

To assess the accuracy of the DL auto-segmentations, metrics were utilized such as the Dice similarity coefficient (DSC), Jaccard coefficient (JC), Hausdorff distance (HD), and average symmetric surface distance (ASSD) with standard deviations (Std) using a Python medpy.metric.binary (Version 0.4.0). Additionally, we evaluated the correlation between the segmentation performance of DSC and liver attenuation using Spearman’s correlation coefficient, as implemented in the Python package scipy.stats (Version 1.5.3).

To compare the DL-based attenuation against the ground-truth attenuation, we performed two-sided Kolmogorov–Smirnov tests and Bland–Altman analyses with the 95% limits of agreements (LOA), utilizing the Python package scipy.stats (Version 1.5.3). Moreover, we assessed the comparison using the mean absolute error (MAE) and intraclass correlation coefficient (ICC) with 95% confidence interval (95% CI), using the statistical software SPSS (Version 25) with a two-way random absolute agreement and single measures approach. To evaluate the performance of steatosis categorization, we calculated the area under the curve (AUC), sensitivity, and specificity using the Python package sklearn.metrics (Version 0.23.2). Furthermore, we employed bootstrap sampling (n = 1000) to estimate the 95% confidence intervals (CI) of the categorization performances.

### Supplementary Information

Supplementary Information.

## Data Availability

Our study materials are collected and reformatted from publicly available datasets under their copyright licenses. Curated CT images and ground truths can be freely downloaded by Google Drive https://drive.google.com/drive/folders/1-g_zJeAaZXYXGqL1OeF6pUjr6KB0igJX or Baidu Wangpan https://pan.baidu.com/s/1nRv-FJU4HtQ4nXi9H9145Q?pwd=2022 (passcode: 2022). The deep learning-based system and the trained nnU-Net model are shared on GitHub (https://github.com/johnnydfci/DL-parenchymal).

## Abbreviations

3D: Three-dimensional
ASSD: Average symmetric surface distance
AUC: Area under the curve
CI: Confidence interval
COVID-19: Coronavirus disease 2019
CT: Computed tomography
DL: Deep learning
DSC: Dice similarity coefficient
HU: Hounsfield unit
HD: Hausdorff distance
ICC: Intraclass correlation coefficient
JC: Jaccard coefficient
LOA: Limits of agreement
MRI: Magnetic resonance imaging
ROI: Region of interest
Std: Standard deviation
US: Ultrasound
