# Supplementary material for: Deep-learning segmentation to select liver parenchyma for categorizing hepatic steatosis on multinational chest CT
Source: Sci Rep. 2024 May 25;14:11987. doi: 10.1038/s41598-024-62887-2 (PMC11127985; doi:10.1038/s41598-024-62887-2)
Supplement: Supplementary file 1 — Supplementary Information. [file 41598_2024_62887_MOESM1_ESM.pdf]

Deep-learning Segmentation to Select Liver Parenchyma for Categorizing Hepatic Steatosis on Multinational Chest CT

Zhongyi Zhang[1], Guixia Li[2], Ziqiang Wang[3], Feng Xia[4], Ning Zhao[5], Huibin Nie[6], Zezhong Ye[7], Joshua Lin[8], Yiyi Hui[9*] & Xiangchun Liu[1*]

1. Department of Nephrology, The Second Hospital of Shandong University, Shandong University, Jinan, 250033, Shandong, China
2. Department of Nephrology, Shenzhen Third People's Hospital, the Second Affiliated Hospital of Southern University of Science and Technology, Shenzhen, 518112, Guangdong, China
3. Department of Nephrology, The First Affiliated Hospital of Hainan Medical University, Haikou, 570102, Hainan, China
4. Department of Cardiovascular Surgery, Wuhan Asia General Hospital, Wuhan, 430000, Hubei, China
5. The First Clinical Medical School, Shanxi Medical University, Taiyuan, 030001, Shanxi, China
6. Department of Nephrology, Chengdu First People's Hospital, Chengdu, 610095, Sichuan, China
7. Independent Researcher, Boston, 02115, MA, USA
8. Keck School of Medicine, University of Southern California, Los Angeles, 90033, CA, USA
9. Department of Medical Imaging, Shandong Provincial Hospital Affiliated to Shandong First Medical University, Jinan, 250021, Shandong, China

* The authors contributed equally to this work

Xiangchun Liu, MD, PhD.

Department of Nephrology, The Second Hospital of Shandong University, Shandong University, Jinan, 250033, Shandong, China

Email: liuxiangchun@sdu.edu.cn

Yiyi Hui, MD.

Department of Medical Imaging, Shandong Provincial Hospital Affiliated to Shandong First Medical University, Jinan, 250021, Shandong, China

Email: huiyiyi@sdfmu.edu.cn

Supplementary Text 1: Dataset licenses and download links

Institutional review board (IRB) oversight was not required for our study, as our materials were collected from publicly available and deidentified datasets [1]. They were originally released in the following publications: LIDC-IDRI [2], NSCLC-Lung1 [3], RIDER [4], VESSEL12 [5], MIDRC-RICORD [6], COVID-19-Italy [7], and COVID-19-China [8]. The individual licenses for each dataset can be found in this supplementary text, along with the direct download links. Accordingly, chest CT images were acquired from these public datasets, and our team of experts conducted manual liver segmentation for the development and validation of our AI methods.

LIDC-IDRI [2]**:** The Image Database Resource Initiative (IDRI) was created to advance the Lung Image Database Consortium (LIDC) in 2004. The data can be accessed here [9]: https://www.cancerimagingarchive.net/collection/lidc-idri/. It's important to note that the accessible images are in the DICOM format. The dataset is licensed under CC BY 3.0 (Creative Commons Attribution License 3.0), allowing various forms of use or re-use as long as due acknowledgment is made of the original source and authorship, with no additional restrictions.

This dataset was studied with approval from its respective licensing institution, along with informed consent from all subjects involved. The initial publication [2] mentioned that these CT scans were gathered and released with the necessary local Institutional Review Board (IRB) approval from the picture archiving and communications systems (PACS) of the seven participating academic institutions.

NSCLC-Lung1 [3]**:** NSCLC stands for non-small cell lung cancer. The Lung1 data set was released in 2014 which consisted of 422 NSCLC patients in the Netherlands. The data can be downloaded here [10]: https://www.cancerimagingarchive.net/collection/nsclc-radiomics/. It

is licensed under CC BY-NC 3.0 (Creative Commons Attribution License 3.0), permitting various forms of use or re-use for non-commercial purposes, provided that due acknowledgment is made of the original source and authorship.

This dataset was studied with approval from its respective licensing committees, along with informed consent from all subjects involved. As stated in the initial publication [3], this public dataset received approval from the Institutional Review Boards of all participating centers, with specific approval from the trial committee at Maastricht University Medical Center (MUMC+) in Maastricht, The Netherlands.

RIDER [4]**:** The RIDER data set consists of 31 patients with two CT scans acquired approximately 15 min apart. The chest CT images can be accessed here [11]: https://www.cancerimagingarchive.net/analysis-result/rider-lungct-seg/. The dataset is licensed under CC BY 3.0 (Creative Commons Attribution License 3.0), allowing various forms of use or re-use as long as due acknowledgment is made of the original source and authorship, with no additional restrictions.

VESSEL12 [5]**:** This dataset is collected from the VESsel SEgmentation in the Lung (VESSEL12) challenge held in 2012. The chest CT images can be accessed through the official challenge website (https://vessel12.grand-challenge.org/) or downloaded from the Kaggle dataset page (https://www.kaggle.com/datasets/andrewmvd/lung-vessel-segmentation). This dataset was released to the public with approval from its respective licensing committees and/or institutions, along with informed/ waived consent from all subjects involved.

As indicated in the initial publication [5], the scans utilized for this challenge were sourced from the anonymized image repositories of three hospitals: University Medical Center Utrecht (Utrecht, The Netherlands), the University Clinic of Navarra (Pamplona, Spain), and

Radboud University Nijmegen Medical Centre (Nijmegen, The Netherlands). In instances where institutional ethics committee approval was mandated, written consent for retrospective studies had been previously acquired from each participant.

MIDRC-RICORD [6]**:** Medical Imaging Data Resource Center (MIDRC); RSNA International COVID-19 Open Radiology Database (RICORD); This set included two subsets, which are Release-1A: Chest CT COVID Positive (MIDRC-RICORD-1a) and Release-1B: Chest CT COVID Positive (MIDRC-RICORD-1b).

The MIDRC-RICORD-1a can be downloaded here [12]: https://www.cancerimagingarchive.net/collection/midrc-ricord-1a/. The MIDRC-RICORD-1b can be downloaded here [13]: https://www.cancerimagingarchive.net/collection/midrc-ricord-1b/. The dataset is licensed under CC BY-NC 4.0 (Creative Commons Attribution License 4.0), permitting various forms of use or re-use for non-commercial purposes, provided that due acknowledgment is made of the original source and authorship.

This dataset was studied with approval from its respective licensing committees and/or institutions, along with informed/ waived consent from all subjects involved. As indicated in the initial publication [6], institutional review board (ethics committee) approval was obtained from all sites for this retrospective study. For the United States site, a waiver of informed consent was obtained, and processes were compliant with the Health Insurance Portability and Accountability Act. Moreover, this public dataset is open for non-commercial use, spanning research, education, and AI system development for various disease entities beyond COVID-19 pneumonia. Hence, we utilized this data for the AI assessment of hepatic steatosis in our study.

COVID-19-Italy [7]**:** The dataset is originally made of 62 COVID-19-positive patients and then enriched to 81 patients. Chest CT images can be downloaded here:

https://www.imagenglab.com/newsite/covid-19/. As stated in the initial publication [7], the dataset is released and licensed under CC BY-NC 4.0 (Creative Commons Attribution License 4.0), permitting various forms of use or re-use for non-commercial purposes, provided that due acknowledgment is made of the original source and authorship. The image collection was conducted with approval from the Hospital Ethics Committee, under the protocol number "Prot. 308," as stated in the initial publication.

COVID-19-China [8]**:** The dataset is made of 29 COVID-19-positive Chinese patients in Hubei Province, China. Chest CT images can be downloaded here, referred to as 'the second dataset [14]: https://www.imagenglab.com/newsite/covid-19/. The dataset is licensed under CC BY 4.0 (Creative Commons Attribution License 4.0), allowing various forms of use or re-use as long as due acknowledgment is made of the original source and authorship, with no additional restrictions.

This dataset was studied with approval from its respective licensing committees and/or institutions, along with informed/ waived consent from all subjects involved. The study [8], conducted at Xiangyang NO.1 People's Hospital Affiliated to Hubei University of Medicine in Xiangyang, Hubei, China, and the University of Milan (Universita Degli Studi Di Milano) Research Board/Institutional Review Board (IRB) in Milan, Italy, received approval (#20200702150947, #324-2020, 562-2020, and #335-2020). The retrospective observational nature of the study led to the waiver of informed consent requirements by both institutions.

Supplementary Text 2: Participants and CT image details in public datasets

LIDC-IDRI [2]**:** The Image Database Resource Initiative (IDRI) was created to further advance the Lung Image Database Consortium (LIDC) in 2004. The LIDC-IDRI contains a total of 1018 chest CT scans from 1010 patients, including both contrast-enhanced and non-enhanced CT scans. Images were collected from 7 participating academic institutions and 8 medical imaging companies in the USA. LIDC consists of diagnostic and lung cancer screening chest CT scans with annotated lung lesions. It is originally used to develop automated lung cancer detection and diagnosis.

LIDC images were constructed from 4 scanner manufacturers and 17 different CT imaging models. The tube peak potential energies used for scan acquisition ranged from 120 to 140 kV. Tube current ranged from 40 to 627 mA. Slice thicknesses ranged from 0.6 to 4.0 mm. The reconstruction interval ranged from 0.45 to 5.0 mm. The in-plane pixel size ranged from 0.461 to 0.977 mm. Each CT scan was initially presented at a standard brightness/contrast setting without magnification. No participant demographics (age, gender, etc.) or clinical information is available for this dataset.

NSCLC-Lung1 [3]**:** NSCLC stands for non-small cell lung cancer. The Lung1 data set was released in 2014 which consisted of 422 NSCLC patients in the Netherlands. 132 are women and 290 are men. The mean age was 67.5 years (range: 33–91 years). Patients were included if they have confirmed diagnoses of lung cancer or underwent treatment with curative intent. This dataset was initially proposed to assess the prognostic value of radiomic features for lung cancer. CT scans and clinical data were available in this study.

RIDER [4]**:** The RIDER data set consists of 31 patients with two CT scans acquired approximately 15 min apart. Patients with non-small cell lung cancer were recruited in 2007 at Memorial Sloan-Kettering Cancer Center, New York, USA. The mean age is 62.1 years

(range, 29-82 years), 16 were men (mean age, 61.8 years; range, 29-79 years) and 16 were women (mean age, 62.4 years; range, 45-82 years). Parameters for the 16-detector row scanner were as follows: tube voltage, 120 kVp; tube current, 299-441 mA; detector configuration. Parameters of the 64-detector row scanner were as follows: tube voltage, 120 kV; tube current, 298-351 mA.

VESSEL12 [5]**:** This dataset is collected from the VESsel SEgmentation in the Lung (VESSEL12) challenge held in 2012, which is to compare automatic methods of lung vessel segmentations taken from both healthy and diseased populations. CT scans were collected from three hospitals in the Netherlands and Spain in a variety of clinically common scanners and protocols. The dataset released 20 CT scans and around 10 scans contain abnormalities such as emphysema, nodules, or pulmonary embolisms.

MIDRC-RICORD [6]**:** Medical Imaging Data Resource Center (MIDRC); RSNA International COVID-19 Open Radiology Database (RICORD); This set included two subsets in April 2020, which are Release-1A: Chest CT COVID Positive (MIDRC-RICORD-1a) and Release-1B: Chest CT COVID Positive (MIDRC-RICORD-1b). Each dataset consists of 120 chest CT scans from four international sites: the USA, Turkey, Canada, and Brazil. The dataset has two inclusion criteria: 1. Adults underwent chest CT scans for suspected COVID-19 infection; 2. COVID-19 positive (1A) confirmed by one or more conditions: reverse-transcription polymerase chain reaction test, immunoglobulin M antibody test, or clinical diagnosis using hospital-specific criteria.

COVID-19-Italy [7]**:** The dataset is originally made of 62 COVID-19-positive patients and then enriched to 81 patients. The group of 62 patients underwent non-contrast chest CT scans in Italy in 2020. The average age was 56 years (range 20–83), and the male/female ratio was 23/27. Images were obtained with two different scanners with reconstructions of the volume at 0.3 to 1 mm slice thickness. Automatic lung tissue classification, clinical score, and

intensive care unit information are provided as well. We chose the enriched set with one CT scan per patient, therefore adding up to 81 CT images in our study.

COVID-19-China [8]**:** The dataset is made of 29 COVID-19-positive Chinese patients who received multiple non-contrast chest CTs between January 21st and April 12th, 2020 in Hubei Province, China. The patients were predominantly female (69%, 20/29), and were 41 ± 10 years old (range 25 to 60 years old). Each patient underwent multiple CT scans at different time points. We chose the baseline CT scans per patient and therefore added up to 29 CT images in our study.

Supplementary Text 3: Implementation details of 3D U-Net

The 3D U-Net model was added with residual connections compared to the original U-Net structure. The input dimension was 64^3 and the output dimension was $2 * 64^3$. Convolutional layers with a stride of 2 were utilized to increase and decrease each stage's dimensions. The kernel's size was 3 * 3 * 3 in all convolutional layers. In addition, parametric rectified linear units and batch normalization were adopted in each down-sampling and up-sampling step. The dice loss function was adopted for the backpropagation algorithm. The 3D U-Net model was implemented in a Linux workstation with an RTX 3060 GPU using Pytorch (version 1.6.0) and MONAI framework (version 0.4.0).

The 3D U-Net model was implemented for comparison in two ways: with or without retraining. Without retraining, the 3D U-Net model was initially developed on 551 non-enhanced chest CT images. These CT images were randomly selected from the National Lung Screening Trial (NLST), which was a randomized controlled trial for lung cancer screening. The LIDC-IDRI set was used to retrain the model, which consisted of 479 CT images and a split of 4:1:1 for training, tuning, and internal testing purposes.

Supplementary Text 4: Outlier analysis of liver segmentation

As the result of nnU-Net segmentation, 91.87% of the DSC values were greater than 0.95, with only 1.46% of DSC being less than 0.90. Most outliers are over-segmented: automated segmentations are bigger than the real liver due to the incorrect inclusion of the spleen or stomach. This might attribute to the different imaging parameters and image qualities. Even when the segmentation DSC is below 0.90, the liver contours are still recognized. The DSC histogram and incorrect segmentations were shown in Figure S4.

Supplementary Text 5: Outlier analysis of DL-parenchymal attenuation measurement

The 95% limits of agreement (LOA) of DL-parenchymal error are from -7.033 to 5.410 and only 5% was out of this LOA. Most outliers were of the sampling errors even though DL correctly selected the parenchymal portion. Of note, incorrect segmentation was not the main reason for attenuation outliers since DL-parenchymal regions were placed correctly away from non-parenchymal regions in most segmentation outliers. The histogram of ground-truth attention and DL-parenchymal attention were shown in Figure S6 along with outlier examples.

Supplementary Tables and Figures

Supplementary Table S1: Data characteristics

Dataset	Partition	CT images / participants	Screening purpose	Location
LIDC-IDRI	Training	319 / 319	Lung cancer	USA
	Tuning	80 / 80		
	Internal test	80 / 80		
Lung1	External test	238 / 238	Lung cancer	Netherlands
RIDER	External test	57 / 29	Lung cancer	USA
VESSEL12	External test	12 / 12	Lung vessel segmentation	Netherlands, Spain
MIDRC-RICORD	External test	128 / 128	Covid-19	USA, Canada, Turkey, Brazil
Covid-19-Italy	External test	71 / 71	Covid-19	Italy
Covid-19-china	External test	29 / 71	Covid-19	China

Table S1: Data characteristics. A total of 1,014 CT images of 986 patients were included in this study. Three datasets were initially obtained to diagnose COVID-19, three datasets were scanned for lung cancer detection, and one dataset was released for lung vessel segmentation. Among these images, 774 (76.3%) were scanned for lung cancer and 228 (22.5%) for COVID-19 respectively.

Supplementary Table S2: Deep learning-based classification of hepatic steatosis with 95% CI.

Metric	Test set	DL-parenchymal	DL-volumetric	DL-axial
AUC	Internal set (n = 80)	0.994 (0.980 – 1.000)	1.000 (1.000 – 1.000)	1.000 (1.000 – 1.000)
	External set (n = 535)	**0.942 (0.899 - 0.975)**	0.937 (0.891 - 0.975)	0.927 (0.879 - 0.964)
Sensitivity	Internal set (n = 80)	1.000 (1.000 – 1.000)	1.000 (1.000 – 1.000)	1.000 (1.000 – 1.000)
	External set (n = 535)	**0.902 (0.815 - 0.966)**	0.885 (0.793 - 0.960)	0.869 (0.772 - 0.944)
Specificity	Internal set (n = 80)	0.987 (0.960 – 1.000)	1.000 (1.000 – 1.000)	1.000 (1.000 – 1.000)
	External set (n = 535)	0.983 (0.971 - 0.994)	**0.989 (0.980 - 0.998)**	0.985 (0.974 - 0.996)

Table S2: Deep learning-based classification of hepatic steatosis with 95% CI. The moderate-to-severe steatosis was rated using DL-based attenuation and presented in AUC, sensitivity, and specificity. Bootstrap sampling (n=1000) was used to derive the 95% CI. AUC = the area under the curve, CI = confidence interval, DL = deep learning.

Supplementary Figure S1: Selection flowchart of CT images

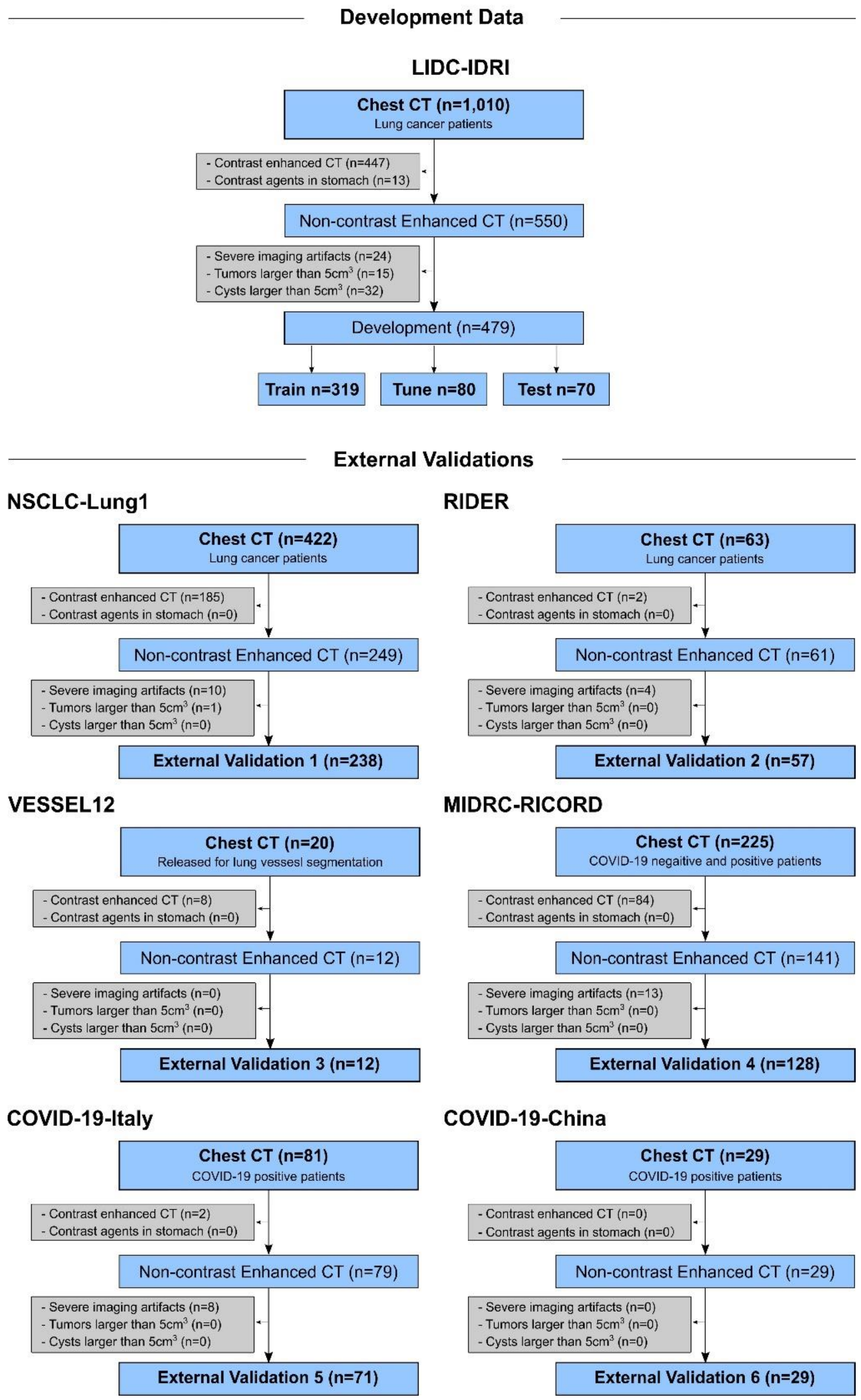

Figure S1. Selection flowchart of CT images. The study used 479 CT images from the LIDC-IDRI dataset for development and 535 CT images from external datasets for external validations.

Supplementary Figure S2: Segmentation performance of nnU-Net vs. U-Net.

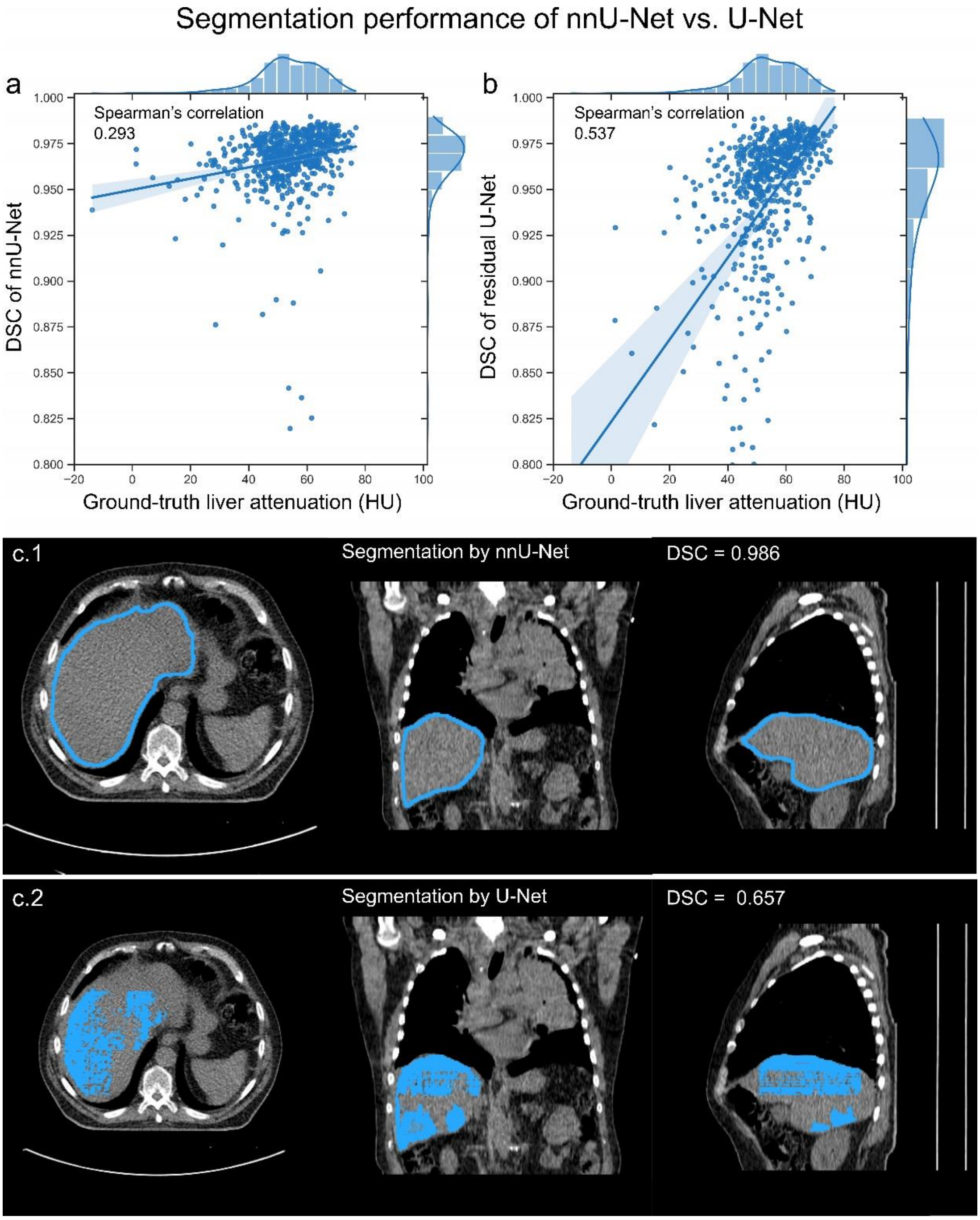

Figure S2: **Segmentation performance of nnU-Net vs. U-Net.** A nnU-Net model and a U-Net model were evaluated for liver segmentation on all test CT images (n=615). The scatter plot presents the Spearman correlation between segmentation DSC and ground-truth attenuation. Segmentation examples showed the superior segmentation of the nnU-Net over the U-Net model. DSC = dice coefficient, HU = Hounsfield unit.

Supplementary Figure S3: Deep learning liver segmentation examples and outliers.

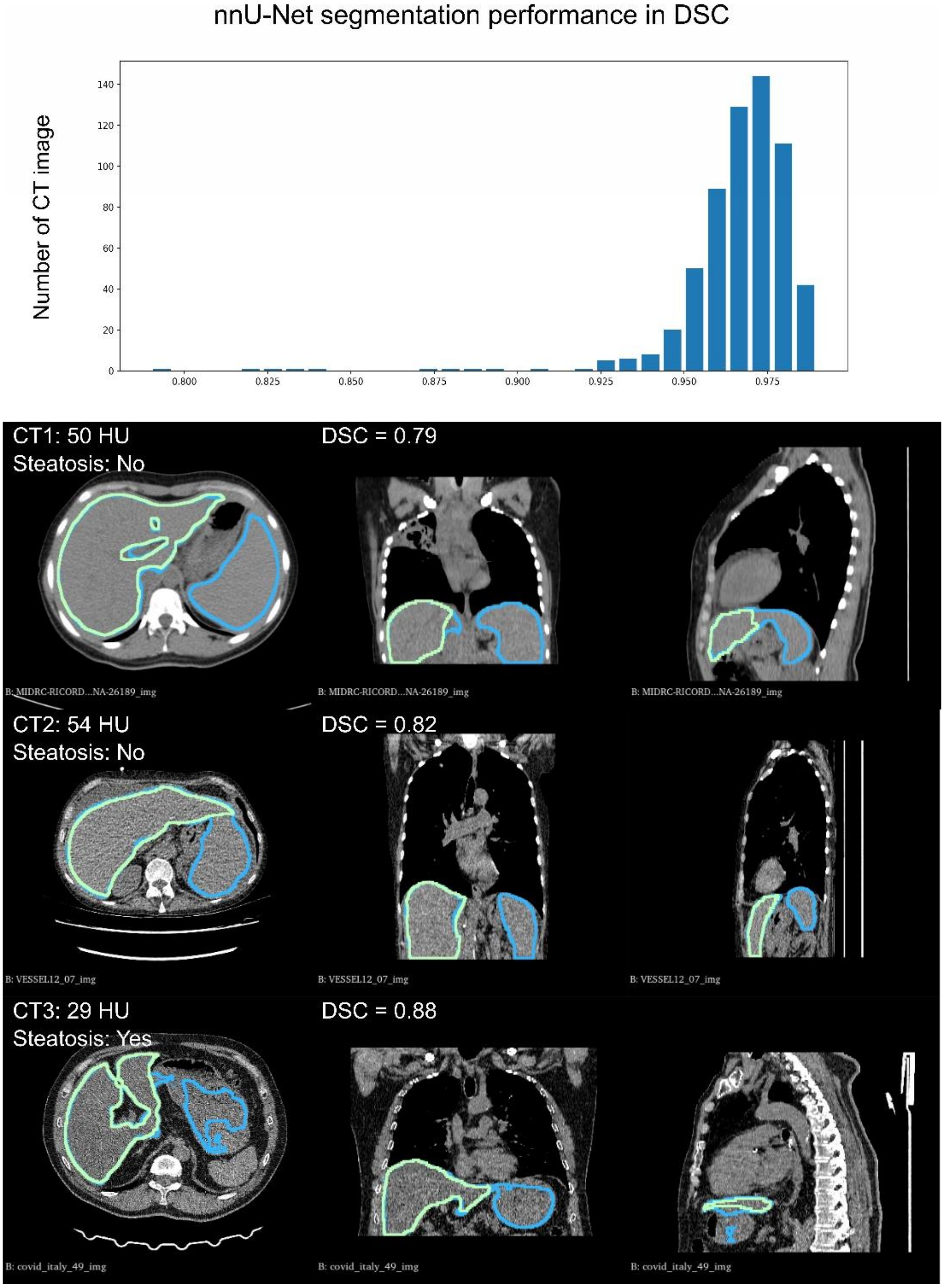

Figure S3: **Automatic liver segmentation examples and outliers.** A nnU-Net model was developed for liver segmentation and was tested on 615 CT images. The DSC histogram and three outliers are shown here. Light green denotes the manual segmentation and blue denotes the automated segmentation. DSC = Dice Coefficient, HU = Hounsfield unit.

Supplementary Figure S4. Accuracy of DL-parenchymal attenuation

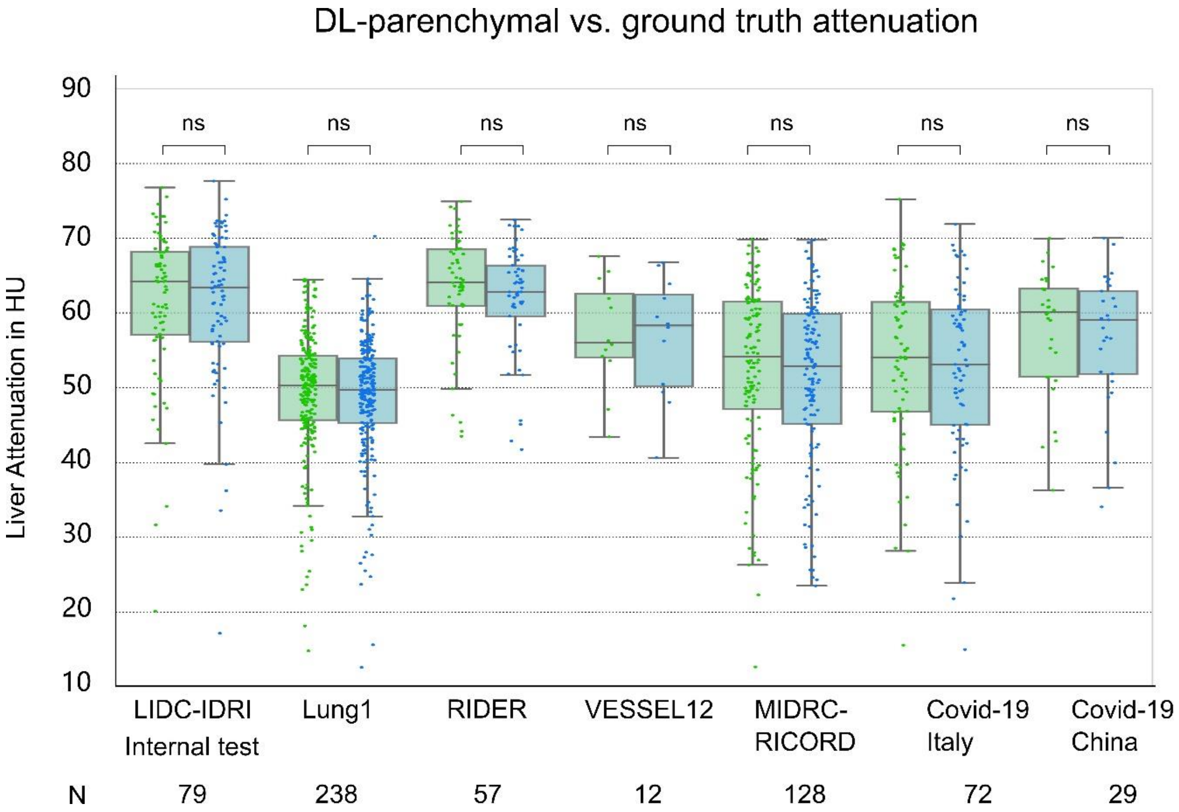

Figure S4. Accuracy of DL-parenchymal attenuation: Figure depicting attenuation measurements of radiologist-validated ground truths (green) vs. DL-parenchymal attenuation (blue). A two-sided Kolmogorov-Smirnov test was used, with a two-tailed p-value indicating significance. ***, $p < 0.001$; **, $p < 0.01$; *, $p < 0.05$. DL = deep learning.

Supplementary Figure S5: Experts vs. DL-parenchymal attenuation

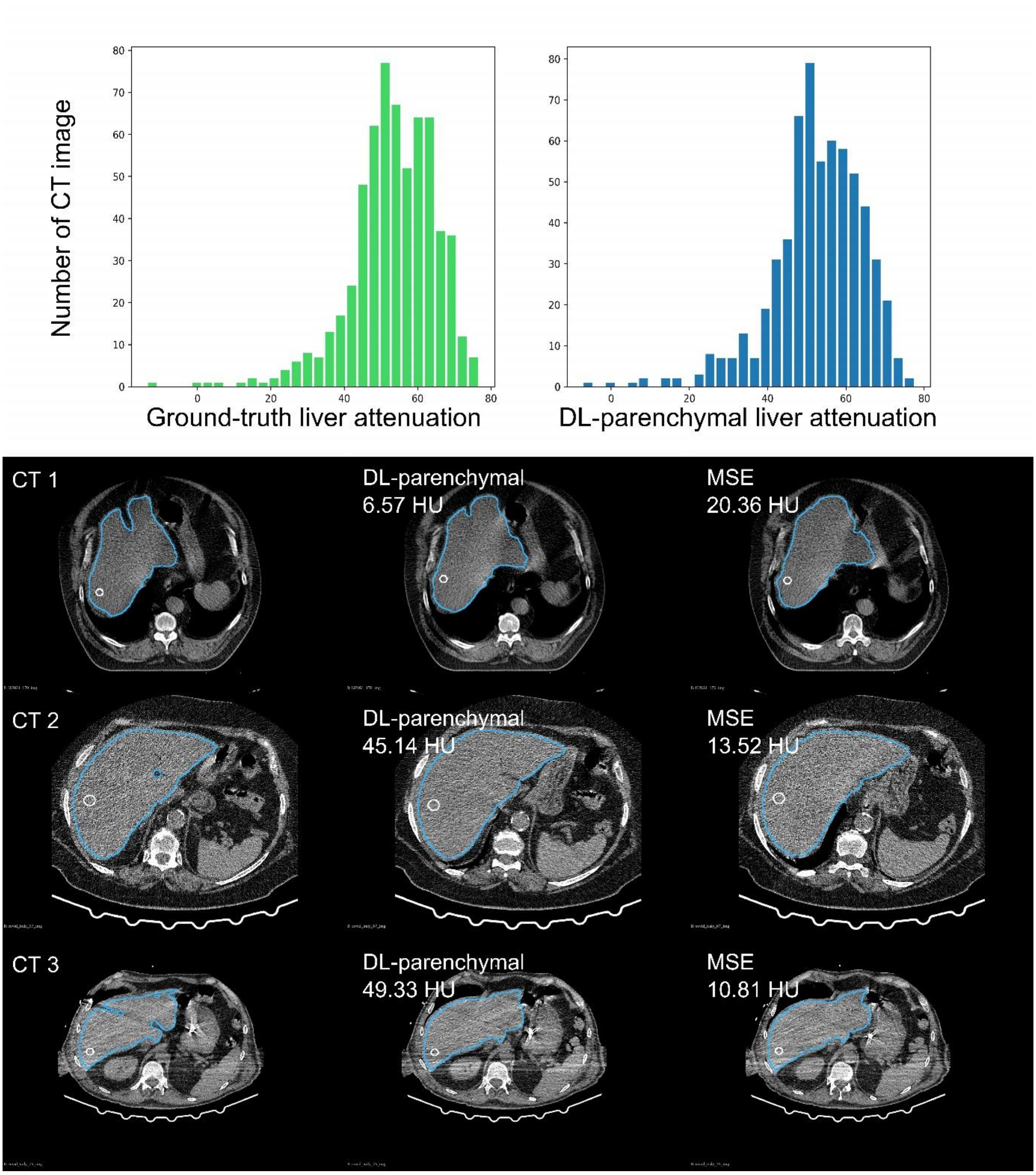

Figure S5: **Experts vs. DL-parenchymal attenuation.** The attenuation of the 615 test CT images was measured by human experts (green) and our DL-parenchymal method (blue), as depicted in histograms. Three examples presented outliers of DL-parenchymal attenuation with DL-based liver segmentation. CT = computed tomography, DL = deep learning, HU = Hounsfield unit, MSE = mean absolute error.

Supplementary Figure S6: Confusion matrices of steatosis classification.

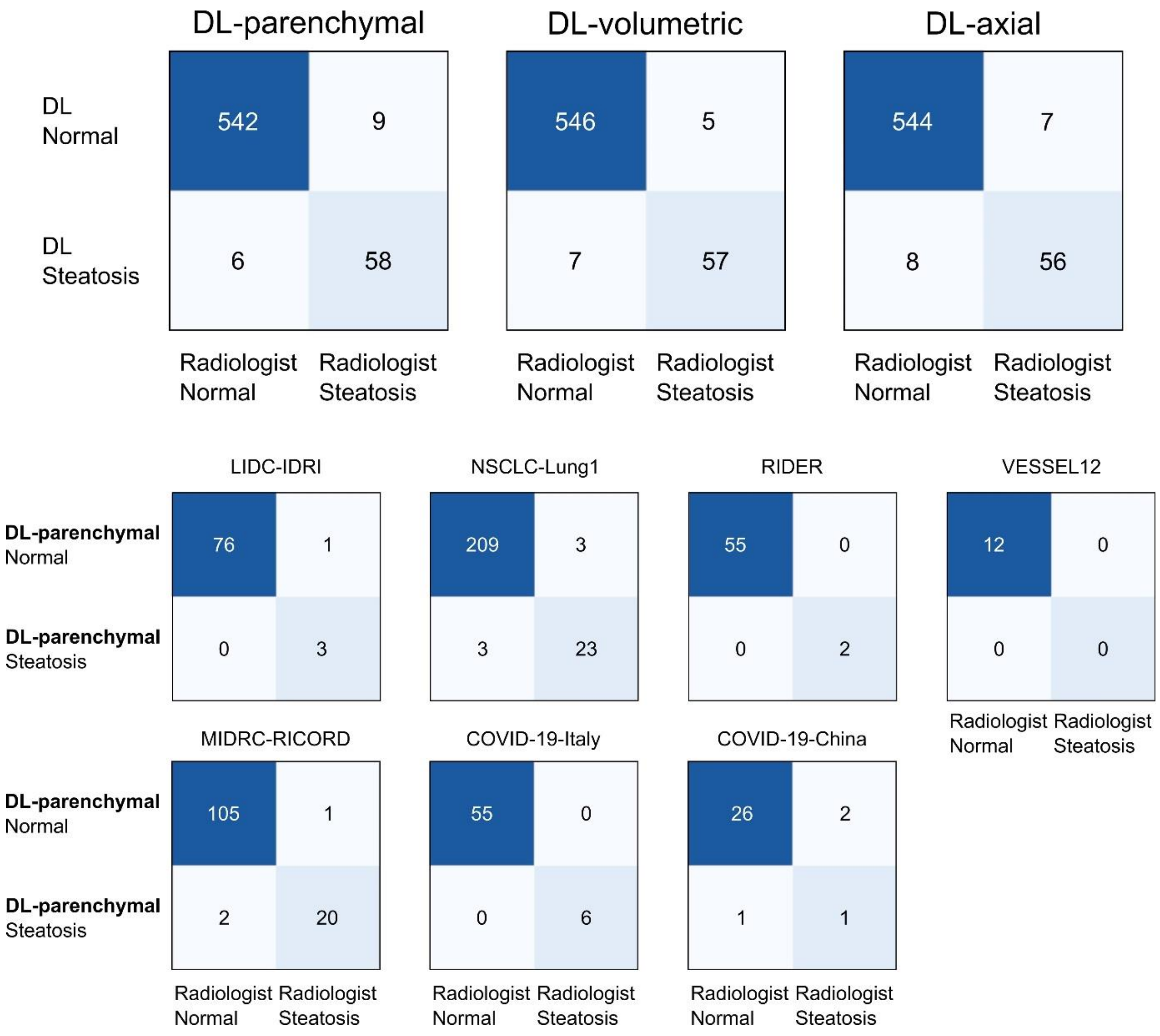

Figure S6: Confusion matrices of hepatic steatosis classification. Three DL-based methods were used to identify moderate-to-severe hepatic steatosis on test sets. The DL-parenchymal method yielded the best classification performance. DL= deep learning.

References

1. Office for Human Research Protections (OHRP). 45 CFR 46. *Hhs.gov* https://www.hhs.gov/ohrp/regulations-and-policy/regulations/45-cfr-46/index.html (2016).
2. Armato, S. G., 3rd *et al.* The Lung Image Database Consortium (LIDC) and Image Database Resource Initiative (IDRI): a completed reference database of lung nodules on CT scans. *Med. Phys.* **38**, 915–931 (2011).
3. Aerts, H., Velazquez, E. R. & Leijenaar, R. T. H. Decoding tumour phenotype by noninvasive imaging using a quantitative radiomics approach. *Nature Communications* **5**, 4006 (2014).
4. Zhao, B. *et al.* Evaluating variability in tumor measurements from same-day repeat CT scans of patients with non-small cell lung cancer. *Radiology* **252**, 263–272 (2009).
5. Rudyanto, R. D. *et al.* Comparing algorithms for automated vessel segmentation in computed tomography scans of the lung: the VESSEL12 study. *Med. Image Anal.* **18**, 1217–1232 (2014).
6. Tsai, E. B. *et al.* The RSNA International COVID-19 Open Radiology Database (RICORD). *Radiology* **299**, E204–E213 (2021).
7. Zaffino, P. *et al.* An Open-Source COVID-19 CT Dataset with Automatic Lung Tissue Classification for Radiomics. *Bioengineering (Basel)* **8**, (2021).
8. Kassin, M. T. *et al.* Generalized chest CT and lab curves throughout the course of COVID-19. *Sci. Rep.* **11**, 6940 (2021).
9. Armato, S. G., III *et al.* Data From LIDC-IDRI. The Cancer Imaging Archive https://doi.org/10.7937/K9/TCIA.2015.LO9QL9SX (2015).
10. Aerts, H. J. W. L. *et al.* Data From NSCLC-Radiomics. The Cancer Imaging Archive https://doi.org/10.7937/K9/TCIA.2015.PF0M9REI (2019).
11. Wee, L., Aerts, H., Kalendralis, P. & Dekker, A. RIDER Lung CT Segmentation Labels from: Decoding tumour phenotype by noninvasive imaging using a quantitative

radiomics approach. The Cancer Imaging Archive https://doi.org/10.7937/TCIA.2020.JIT9GRK8 (2020).

12. Tsai, E. *et al.* Medical Imaging Data Resource Center - RSNA International COVID Radiology Database Release 1a - Chest CT Covid+ (MIDRC-RICORD-1a). The Cancer Imaging Archive https://doi.org/10.7937/VTW4-X588 (2020).
13. Tsai, E. B. *et al.* Medical Imaging Data Resource Center (MIDRC) - RSNA international COVID open research database (RICORD) release 1b - chest CT covid-. The Cancer Imaging Archive https://doi.org/10.7937/31V8-4A40 (2021).
14. An, P. *et al.* CT Images in COVID-19. The Cancer Imaging Archive https://doi.org/10.7937/TCIA.2020.GQRY-NC81 (2020).
